# The α-Glucosidase Inhibition Activities of Phaeochromycins D and E Isolated from Marine *Streptomyces* sp. FJ0218

**DOI:** 10.3390/molecules30091993

**Published:** 2025-04-30

**Authors:** Pingfa Lin, Mianmian Shi, Feifei Wang, Yong Lin, Yongbiao Zheng

**Affiliations:** 1 School of Pharmacy, Fujian Health College, Fuzhou 350101, China; linpingfa@163.com; 2 College of Life Sciences, Fujian Normal University, Fuzhou 350117, China

**Keywords:** phaeochromycins, α-glucosidase, *Streptomyces* sp.

## Abstract

Marine *Streptomyces* are an important source of naturally occurring active compounds. Out of 23 marine *Streptomyces* strains, 1 strain of *Streptomyces* sp. FJ0218 was selected for its high activity in inhibiting α-glucosidase. Two polyketides, phaeochromycins D (**2**) and E (**1**), were isolated from the fermentation broth of this strain using bioactivity-guided column chromatography over RP-18, Sephadex LH-20, and silica gel. Their structures were determined using NMR data, HR-EI-MS, and single-crystal X-ray crystallography. Phaeochromycins D (**2**) and E (**1**) exhibited inhibitory activity against α-glucosidase, with IC_50_ values of 10 mM and 25 mM, respectively. Lineweaver–Burk plots revealed that phaeochromycin E (**1**) acts as an uncompetitive inhibitor, while phaeochromycin D (**2**) acts as a non-competitive inhibitor. These findings suggest that there is potential for the pharmacological regulation of glucose levels through the use of polyketide phaeochromycins, emphasizing their significant impact on glucose management.

## 1. Introduction

Diabetes mellitus is a condition characterized by abnormal glucose regulation and high blood sugar levels, which can lead to damage to the heart, eyes, kidneys, and nerves, as well as other complications. According to the World Health Organization, diabetes mellitus is expected to become a top 10 deadliest disease. As of 2017, there were an estimated 425 million cases of diabetes, affecting 8.8% of adults aged 20–79 [1]. It is projected that by 2045, 629 million people, or 9.9% of the 20–79 age group, will have diabetes. The global cost of diabetes was estimated to be USD 760 billion in 2019, and it is projected to increase to USD 825 billion by 2030 and USD 845 billion by 2045 [2]. Given the significant complications and economic burden of diabetes, there has been substantial research on, and the development of, antidiabetic drugs. One target for screening these drugs is α-glucosidase in the upper part of the small intestine. α-Glucosidase inhibitors can delay carbohydrate absorption and regulate postprandial blood glucose levels. Currently, drugs like acarbose, voglibose, and miglitol are approved for lowering blood glucose levels [3,4]. However, the use of these drugs may be limited due to gastrointestinal side effects, such as abdominal distention, diarrhea, and flatulence [5]. Therefore, there is ongoing research for novel α-glucosidase inhibitors with improved efficacy and safety, which is of interest to many pharmacologists.

The ocean features unique ecological environments, such as a high level of salinity, high pressure, a low temperature, and low nutrition. Marine organisms can produce numerous natural products with diverse activities and novel structures [6]. Approximately 70% of these marine natural products are derived from marine actinomycetes, with around 50–55% originating from marine *Streptomyces* [7]. Many natural products from marine *Streptomyces* exhibit unique chemical structures and a wide range of antibacterial, fungal, tumorous, and cytotoxic bioactivities, demonstrating their significant potential in providing important lead compounds for the development of new drugs [8]. In recent years, these findings have garnered an increasing amount of attention. A partially purified fraction of marine *Streptomyces* sp.S2A showed α-glucosidase and α-amylase inhibition with IC_50_ values of 21.17 μg/mL and 20.46 μg/mL, respectively [9]. Some of the secondary metabolites originating from two mangrove-derived marine *Streptomyces* sp. WHUA03267 and *Streptomyces* sp. WHUA03072 exhibited an apparent inhibitory activity against α-glucosidase [10]. In this paper, a strain exhibiting high α-glucosidase inhibitory activity was selected from 23 marine *Streptomyces* strains. Two compounds with α-glucosidase inhibitory activity were obtained through the separation and purification of their metabolites.

## 2. Results

### 2.1. The α-Glucosidase Inhibitory Activity of Organic Crude Extracts from Marine Streptomyces

**Figure 1:**
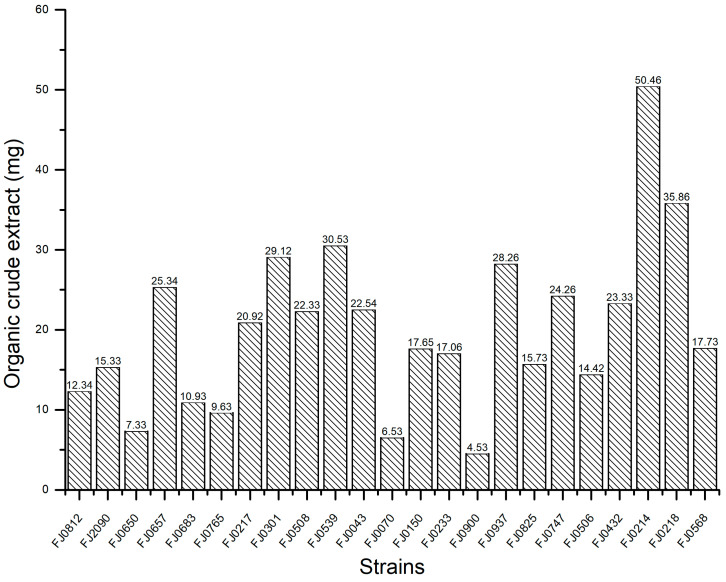
The yields of organic crude extracts from 23 strains of marine *Streptomyces*.

**Figure 2:**
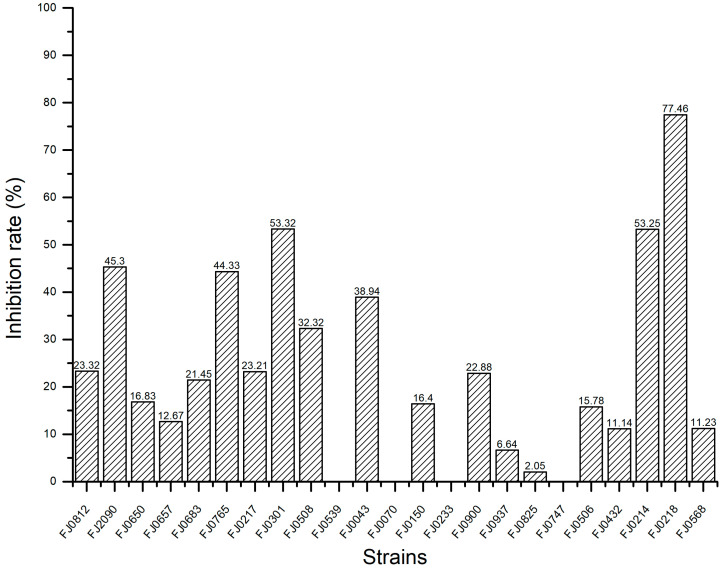
The inhibition rate against α-glucosidase of organic crude extracts from 23 marine *Streptomyces*.

The yields of organic crude extract prepared from 23 strains of marine *Streptomyces* are provided in Figure 1. The α-glucosidase inhibitory activities of these organic crude extracts at a concentration of 5 mg/mL are shown in Figure 2. Our experimental results revealed that the inhibitory effect of these strains against α-glucosidase varied significantly, and one strain, *Streptomyces* sp. FJ0218, exhibited the highest α-glucosidase inhibitory activity with an inhibition rate of 77.5%. As a result, this strain was chosen for further exploration of its active chemical components.

### 2.2. The Liquid Fermentation, Preparation of Extracts, and Purification Processes

The marine *Streptomyces* sp. FJ0218 was chosen for submerged liquid fermentation and produced 3.1 g of organic crude extract. Subsequently, the 1.9 g organic crude extract was divided into 22 fractions using MPLC over RP-C18 silica gel (170 g). The separation was performed using a stepwise gradient of CH_3_OH in H_2_O (*v*/*v* 0:100, 5:100, 30:100, 50:50, 70:100, and 100:0) based on TLC analyses. From these fractions, two fractions were obtained from the gradient mobile phase of the CH_3_OH in H_2_O (*v*/*v* 50:50), namely Fr.1 (109.5 mg) and Fr.2 (61.2 mg). Fr.1 was further subjected to chromatography over Sephadex LH-20 (120 g) and eluted with methanol to yield the subfraction Fr.11 (31 mg). Fr.11 was then purified through silica gel chromatography (1.2 g) using a CHCl_3_–CH_3_OH solvent gradient of 100:1 to yield compound **1** (12.8 mg). Fr.2 was separated via column chromatography over Sephadex LH-20 (120 g) with methanol as the eluant, affording the subfraction Fr.21(18.5 mg). Fr.21 was further separated using column chromatography over silica gel (1 g) using a CHCl_3_–CH_3_OH solvent gradient of 200:1, yielding pure compound **2** (5.4 mg).

### 2.3. Structural Identification

**Figure 3:**
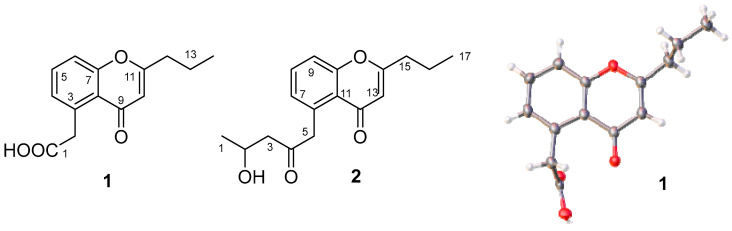
The chemical structures of compounds **1** and **2** and the crystal form of **1** (The CIF data are provided in the Supplementary Materials).

Compound **1** was isolated as a white substance. The ^13^C NMR and DEPT data (Figure S2), recorded in CDCl_3_ at 126 MHz, indicated that compound **1** contains 14 carbon signals: 1 methyl at δ_C_ 13.7 (C-14); 3 methylenes at δ_C_ 20.3 (C-13), 36.0 (C-12), and 41.8 (C-2); 6 *sp*^2^ carbons at δ_C_ 110.8 (C-10), 118.2 (C-6), 121.8 (C-8), 129.1 (C-4), 133.7 (C-5), and 135.0 (C-3); and 3 possible carbonyl carbons at δ_C_ 170.0 (C-11), 173.2 (C-1), and 181.6 (C-9). Eight proton peaks for δ_H_ 7.61 (dd, *J* = 8.5, 7.4 Hz, 1H, H-5); 7.42 (dd, *J* = 8.5, 1.2 Hz, 1H, H-6); 7.28 (d, *J* = 7.4, 1.2 Hz, 1H, H-4); 6.24 (s, 1H, H-10); 4.20 (s, 2H, H-2); 2.62 (o, 2H, H-12); 1.78 (o, 2H, H-13), and 1.03 (t, *J* = 7.4 Hz, 3H, H-14) were observed in the ^1^H NMR spectra (500 MHz, Chloroform-*d*) of **1** (Figure S1). The molecular formula of **1** was determined to be C_14_H_14_O_4_ by HR Q-TOF MS at 247.0961 for [M + H]^+^ (calculated for C_14_H_15_O_4_, 247.0970) and at 269.0781 for [M + Na]^+^ (calculated for C_14_H_14_O_4_Na, 269.0790). Based on the NMR and HR Q-TOF MS data (Figure S3), compound **1** was identified as phaeochromycin E [11], which was confirmed via X-ray diffraction (Figure 3). The crystallographic data for **1** were the following: C_14_H_15_O_4_, triclinic, space group P-1, *a* = 5.1043(2) Å, *b* = 9.9464(4) Å, *c* = 12.0965(5) Å, α = 95.803(3)°, *β* = 95.006(3)°, *γ* = 102.724(3)°, *V* = 592.17(4) Å^3^, *Z* = 2, *Dc* = 1.3810 g/cm^3^, F(000) = 260.9, 10,549 reflections measured (7.4° ≤ 2θ ≤ 143.52°), 2147 unique (*R*_int_ = 0.0736), which were used in all calculations. The final *R*_1_ was 0.0497 (*I* ≥ 2σ (*I*)) and the *wR*_2_ was 0.1401 (all data). The crystallographic data have been deposited with the Cambridge Crystallographic Data Centre (CCDC 2383783).

Compound **2** was isolated as a white substance. The ^13^C NMR and DEPT data (Figure S5), recorded in CDCl_3_ at 126 MHz, revealed that compound **2** exibits 17 carbon signals for 2 methyls at δ_C_ 13.7 (C-17) and 22.7 (C-1); 4 methylenes at δ_C_ 20.3 (C-16), 36.0 (C-15), 50.0 (C-5), and 51.3 (C-3); 1 oxygenating methine at δ_C_ 64.5 (C-2); 6 *sp*^2^ carbons at δ_C_ 111.0 (C-13), 117.9 (C-9), 121.7 (C-11), 128.9 (C-7), 132.9 (C-8), and 135.8 (C-6); and 4 possible carbonyl carbons at δ_C_ 158.1 (C-10), 168.8 (C-14), 179.9 (C-12), and 208.3 (C-4). In the ^1^H NMR (500 MHz, Chloroform-*d*) (Figure S4), the following peaks could be observed at δ 7.56 (dd, *J* = 8.5, 7.3 Hz, 1H, H-8); 7.38 (dd, *J* = 8.5, 1.2 Hz, 1H, H-9); 7.04 (dd, *J* = 7.3, 1.2 Hz, 1H, H-7); 6.06 (s, 1H, H-13); 4.38 (ddd, *J* = 9.3, 6.3, 2.6 Hz, 1H, H-2); 4.33 (d, *J* = 17.0 Hz, 1H, H-5a); 4.19 (d, *J* = 17.0 Hz, 1H, H-5b); 2.86 (dd, *J* = 16.5, 2.6 Hz, 1H, H-3a); 2.76 (dd, *J* = 16.5, 9.3 Hz, 1H, H-3b); 2.56 (o, 2H, H-15); 1.78 (o, 2H, H-16); 1.25 (d, *J* = 6.3 Hz, 3H, H-1); and 1.02 (t, *J* = 7.4 Hz, 3H, H-17). The molecular formula of **2** was determined to be C_17_H_20_O_4_ by HR Q-TOF MS at 289.1434 for [M + H]^+^ (calculated for C_17_H_21_O_4_, 289.1440) and at 311.1253 for [M + Na]^+^ (calculated for C_17_H_20_O_4_Na, 311.1259) (Figure S6). Based on the NMR and HR Q-TOF MS data, compound **2** was identified as phaeochromycin D [11] (Figure 3). Phaeochromycins are a series of aromatic type II polyketides isolated from *S. phaeochromogenes* LL-P018 [11], *Streptomyces* sp. DSS-18 [12], and *Streptomyces* sp. 166 [13]. The type II polyketide synthase cluster *als* in *S. sundarbansensis* SCSIO NS01 was identified as the biosynthesis cluster for phaeochromycin B through genome sequencing and in vivo gene inactivation experiments [14]. The biosynthesis pathways of phaeochromycins B, D, E, and L were proposed via heterologous expression of the *als* cluster. Phaeochromycins B, D, and L are derived from a linear nonaketide that initially cyclizes at the C-7/C12 junction. In contrast, phaeochromycin E is proposed to originate from a heptaketide, which undergoes spontaneous first-ring cyclization at C-3/C-8, followed by a C-7-OH/C-11 cyclization. Phaeochromycin E may also be derived from phaeochromycin D through retro aldol and/or oxidative cleavage [14]. The specific biosynthetic pathway of phaeochromycins remains to be further elucidated through experimental validation.

### 2.4. The α-Glucosidase Inhibitory Activities of Compounds **1** and **2**

**Figure 4:**
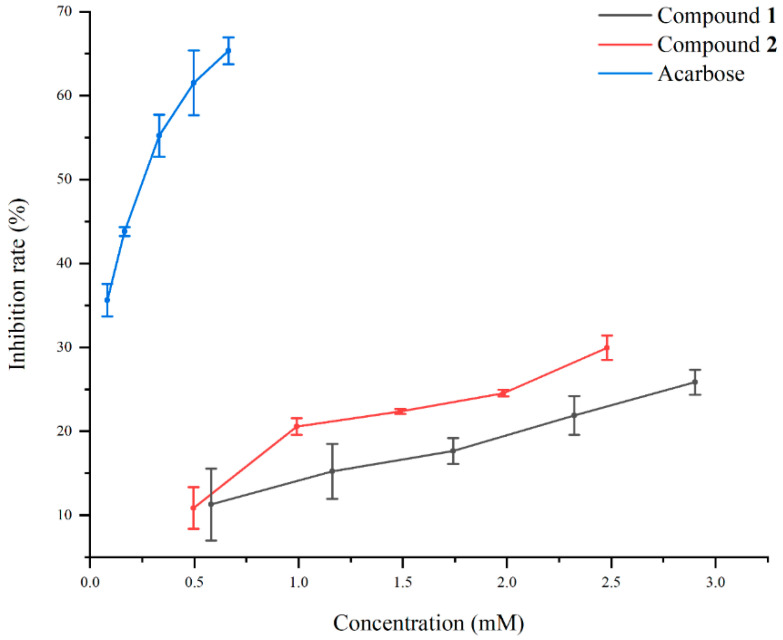
The inhibitory activity against the α-glucosidase of compounds **1** and **2**.

**Figure 5:**
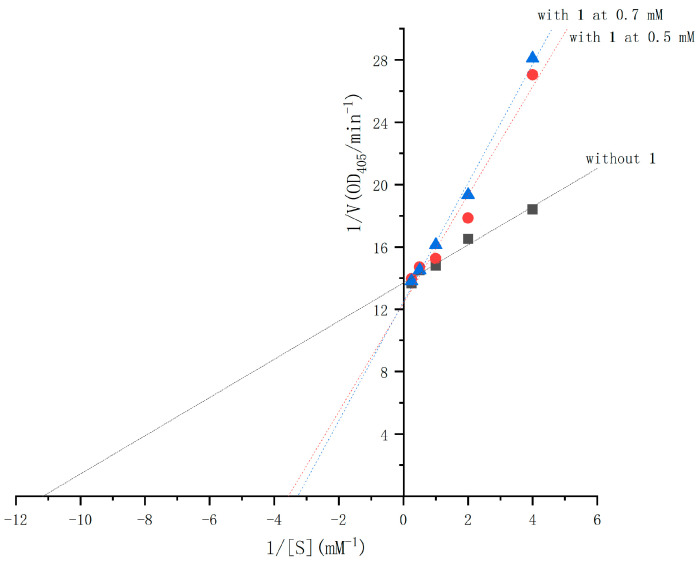
Double-reciprocal plots of the inhibition kinetics of α-glucosidase by compound **1**.

**Figure 6:**
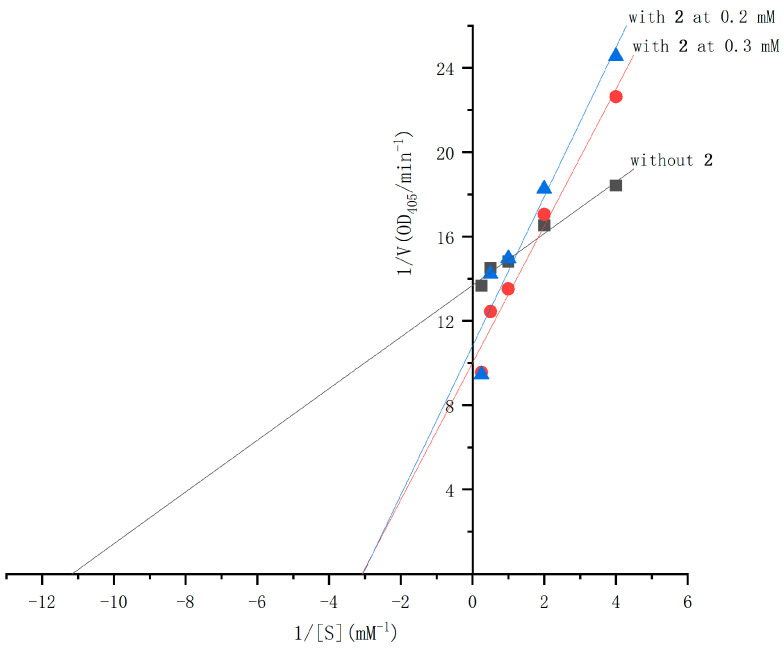
Double-reciprocal plots of the inhibition kinetics of α-glucosidase by compound **2**.

The α-glucosidase inhibitory activities of compounds **1** and **2** were evaluated using the chromogenic method. Both compounds demonstrated dose-dependent inhibition, with IC_50_ values of 25 mM for **1** and 10.3 mM for **2** (see Figure 4), compared to the IC_50_ value of 0.25 mM for acarbose. The inhibition mechanisms of compounds **1** and **2** were analyzed using Lineweaver–Burk plots and the double-reciprocal method to study enzyme kinetics. In the Lineweaver–Burk plot for compound **1** (Figure 5), adding it at 0.5 mM and 0.7 mM increases the vertical intercept 1/*Vmax*, indicating a decrease in *Vmax*, and decreases the absolute value of the horizontal intercept −1/*Km*, meaning *Km* decreases. Compound **1** acts as an uncompetitive inhibitor by binding to the enzyme–substrate complex. For compound **2** (Figure 6), adding it at 0.2 mM and 0.3 mM increases the vertical intercept 1/*Vmax*, decreasing the 1/*Vmax*, while the absolute value of the horizontal intercept −1/*Km* remains unchanged, keeping the *Km* constant. Compound **2** is a non-competitive inhibitor that binds to both the free enzyme and the enzyme–substrate complex at a site different from the substrate-binding site. Mechanistically, compound **1** binds to the enzyme–substrate complex, whereas compound **2** can bind to both the free enzyme and the enzyme–substrate complex at a distinct site. Uncompetitive inhibitors like compound **1** have effects related to substrate concentration, being less effective at low substrate concentrations. Non-competitive inhibitors like compound **2** depend solely on their concentration for inhibition. Visually, compound **2** may exhibit more significant inhibition at the same concentration due to a more apparent drop in the *Vmax*. These results align with the IC_50_ values determined for both compounds.

Currently, α-glucosidase inhibitors are categorized into several chemical structural types, including pseudo-oligosaccharides (e.g., Acarbose and Voglibose), amino-sugars/cyclitols (e.g., 1-Deoxynojirimycin and Miglitol), heterocyclic compounds (e.g., neoponkoranol and pyrimidine derivatives), polyphenolic derivatives (e.g., Raspberry ketone, Gallotannin, and flavonoids), and polysaccharides (e.g., Fucoidan). These inhibitors operate via various mechanisms, such as competitive, non-competitive, and uncompetitive inhibition. Pseudo-oligosaccharide inhibitors mimic the structure of natural substrates like maltose and sucrose, binding competitively to the enzyme’s active site. Aminosugars/cyclitols, resembling glucose, also bind competitively to the active site [15]. Neoponkoranol and its sulfonium salt derivatives exhibit strong competitive inhibition against small intestinal α-glucosidase in rats [16]. Raspberry ketone inhibits α-glucosidase non-competitively, binding reversibly and quickly [17]. Gallotannin shows a parabolic mixed-type inhibition pattern [18]. A pyrimidine derivative, 3-amino-2,4-diarylbenzo[4,5]imidazo[1,2-a]pyrimidine, interacts competitively with key amino acids at the enzyme’s active site [19].

**Figure 7:**
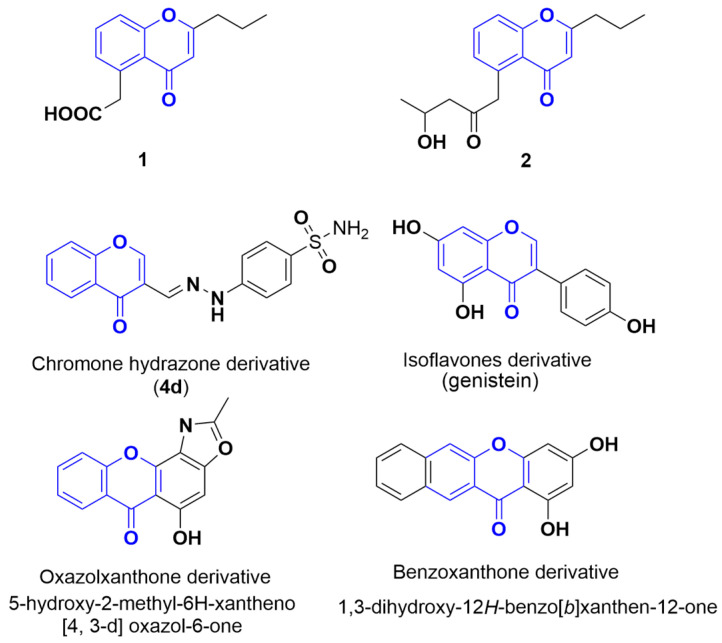
Some α-glucosidase inhibitors with the moiety of chromone (in blue).

Among the reported α-glucosidase inhibitors, compounds with chromone structures show favorable inhibitory activity and distinct mechanisms. A series of chromone hydrazone derivatives were synthesized and demonstrated excellent α-glucosidase inhibition. Compound **4d**, featuring a 4-sulfonamide substitution at the phenyl part of the hydrazide (Figure 7), emerged as the most active compound, exhibiting a non-competitive mode according to the Lineweaver–Burk plot analysis [20]. Certain benzoxanthone derivatives containing chromone and oxazole rings were more potent than 1-deoxynojirimycin. Kinetic analysis revealed that one compound (5-hydroxy-2-methyl-6H-xantheno[4,3-d]oxazol-6-one) (Figure 7) acts as a competitive inhibitor, while others are non-competitive. Additionally, adding electron-donating groups and halogens to the phenyl ring enhances inhibitory activity, whereas electron-withdrawing groups reduce it [21]. Structure–activity correlation analysis indicated that benzoxanthone derivatives (Figure 7) with larger conjugated π-systems and more hydroxyl groups exhibit higher α-glucosidase inhibitory activity [22]. Isoflavones like genistein (Figure 7) and daidzein were also found to be effective α-glucosidase inhibitors with non-competitive action [23].

In this study, phaeochromycins D (**2**) and E (**1**), which both contain chromone moieties, demonstrated α-glucosidase inhibition through different mechanisms. The structure–activity relationship (SAR) analysis of these chromone derivatives revealed that modifying the structures of compounds **1** and **2** could help in developing more potent inhibitors. This provides valuable insights for creating stronger α-glucosidase inhibitors.

Several studies have reported the biological activities of phaeochromycins. Phaeochromycins A and C were found to be weak inhibitors of MAPKAP kinase-2 (IC_50_) 39 and 130 µM, respectively) [11], and phaeochromycin H exhibits a modest inhibitory rate (46.0%) against the HeLa cell line at a concentration of 10 μg/mL [12]. Phaeochromycins J and K exhibited a selective antiproliferative activity against H1299 and HUCCT1 cell lines, with the IC_50_ values ranging from 8.83 to 10.52 μM [13]. This has been the first study to report that phaeochromycins D and E possess inhibitory activity against the α-glucosidase.

## 3. Materials and Methods

### 3.1. Strains Materials

The strains were provided by the Engineering Research Center of Marine Biopharmaceutical Resources, Xiamen Medical College, and the Fujian Institute of Microbiology.

### 3.2. The Liquid Fermentation and Preparation of Extracts of Marine Streptomyces

A total of 23 strains of marine *Streptomyces* were cultured using submerged liquid fermentation. The process was carried out in 250 mL Erlenmeyer flasks containing 100 mL of improved Gauze’s synthetic medium No. 1. The medium consisted of 15 g/L soluble starch, 15 g/L anhydrous glucose, 5 g/L K_2_HPO_4_·3H_2_O, 0.01 g/L FeSO_4_·7H_2_O, 1.0 g/L KNO_3_, and 0.2 g/L MgSO_4_·7H_2_O, with a total volume of 0.5 L. The flasks were then incubated in a constant temperature shaker at 28 °C with a shaking speed of 210 rpm for 25 days. After incubation, the culture broth was centrifuged at 5000 rpm for 30 min to remove the mycelia. The resulting liquid was then extracted with ethyl acetate at a 1:1 ratio. The organic phase was dehydrated by adding an appropriate amount of anhydrous sodium sulfate and concentrated under reduced pressure at 40 °C to yield the organic crude extract of these strains. These organic crude extracts were subjected to an assay for α-glucosidase inhibitory activity. Subsequently, one strain exhibiting a high inhibition rate on α-glucosidase inhibitory activity was selected to explore the active chemical components. This strain was cultured with a total volume of 45 L using the above methods. Then the organic crude extract was obtained with the same process.

### 3.3. Purification and Component Separation

Column chromatography was performed using RP-18 (Merck, Darmstadt, Germany), Sephadex LH-20 (Amersham Biosciences, Uppsala, Sweden), and silica gel (Qingdao Marine Chemical Company, Qingdao, China) to retrieve active chemical constituents from the organic crude extract through bioactivity-guided fractionation.

### 3.4. Structural Characterization

The chemical structures were determined using NMR data, HR-QTOF-MS, and single-crystal X-ray crystallography. The NMR spectra were obtained using a Bruker AVANCE III 500 spectrometer (Bruker, Billerica, MA, USA) operating at 500/126 MHz. HR-QTOF-MS spectra were recorded on an Agilent 6520 mass spectrometer (Agilent Technologies Inc., Santa Clara, CA, USA) in the positive mode (4.0 KV) across the mass range *m*/*z* 200 to 800. A suitable crystal of 1 obtained from aqueous acetone was selected and analyzed using an Oxford Gemini S Ultra diffractometer (Oxford Instruments, Oxfordshire, UK) with the Cu-Kα (λ = 1.54184 Å) radiation at 273 K. The crystal structures were determined using the direct method and refined with full-matrix least-squares calculations on *F*^2^ using olex2-2.1 [24].

### 3.5. α-Glucosidase Inhibitory Activity

The α-glucosidase inhibitory activity was determined using the chromogenic method as described by Walker [25] with some modifications. First, 100 U of α-glucosidase (G5003, Sigma, St. Louis, MO, USA) was dissolved in 1 mL of 100 mM potassium phosphate buffer (PBS, pH 6.8) containing 0.2% BSA, and then diluted to make 0.2 U/mL of an enzyme solution for the next assay. A substrate solution was made using 2.5 mM of ρ-nitrophenyl-α-D-glucopyranoside (ρNPG, N1337-1G, Sigma, St. Louis, MO, USA) in the same PBS buffer. Next, 140 μL of 100 mM PBS, 20 μL of 0.2 U/mL α-glucosidase, and 20 μL of 5 mg/mL of the test sample were added to a 96-well plate. After being thoroughly mixed, the mixture was placed in a 37 °C constant temperature incubator for 15 min. Following incubation, 20 μL of 2.5 mM ρNPG was added and incubated at 37 °C for another 15 min. Then, 80 μL of 200 mM Na_2_CO_3_ solution was added to complete the reaction. Finally, the absorbance value (OD) was measured at 405 nm using a microplate reader. The experiment used 20 μL of PBS as the background control instead of 20 μL of α-glucosidase solution. Additionally, 20 μL of PBS was used as the negative control instead of the test sample. The positive control consisted of 20 μL of 1.5 mg/mL acarbose (117063, Bayer, Leverkusen, Germany) solution. Each treatment was repeated three times. The α-glucosidase inhibitory activity was calculated using the following formula: Inhibitory rate (%) = [(An − As)/An] × 100, where As represents the absorbance of the sample reaction solution, and An represents the absorbance of the negative control. The IC_50_ values, the inhibitor concentration in mM that inhibits 50% of the enzyme activity, were determined graphically by interpolating from the inhibitions determined with different concentrations of compounds, through statistical analysis using SPSS 18.0.

The inhibition types of compounds **1** and **2** were determined using Lineweaver–Burk plots, following methods similar to those previously reported. For compound **1**, concentrations of 0.5 mM and 0.7 mM were used, while for compound **2**, concentrations of 0.2 mM and 0.3 mM were selected. With each concentration, a-glucosidase activity was assayed by varying the concentration of ρNPG (0.25 mM, 0.50 mM, 1.0 mM, 2.0 mM, and 4.0 mM). The enzyme reaction was performed using the conditions described above. Inhibition types were analyzed using double-reciprocal plots generated with Origin 2021.

## 4. Conclusions

The category of natural products with α-glucosidase inhibitory activity includes a variety of chemical structures, such as acarbose, voglibose, stilbenoids [26,27], flavonoids [28], and anthocyanidins [29]. This paper has discussed the discovery of polyketide phaeochromycins as potent α-glucosidase inhibitors for the first time. While phaeochromycins E and D require extensive follow-up studies, including structural modifications, in vivo validation, cytotoxicity assays, and evaluations of drug-likeness, they still offer new chemical resources for researching and developing treatments for diabetes.

## Data Availability

All data are within this manuscript.

## Supplementary Materials

The following supporting information can be downloaded at: https://www.mdpi.com/article/10.3390/molecules30091993/s1, File S1: Figures S1–S6: ^1^H NMR, ^13^C NMR, and HR Q-TOF MS data for compounds **1** and **2**; File S2: The CIF data of compound **1** (CCDC-2383783).
