# Supplementary material for: The α-Glucosidase Inhibition Activities of Phaeochromycins D and E Isolated from Marine Streptomyces sp. FJ0218
Source: Molecules. 2025 Apr 30;30(9):1993. doi: 10.3390/molecules30091993 (PMC12073238; doi:10.3390/molecules30091993)
Supplement: Supplementary file 1 [file molecules-30-01993-s001.zip › File S1.pdf]

Electronic Supplementary Information

The α-Glucosidase Inhibition Activities of Phaeochromycins D and E Isolated from Marine *Streptomyces* sp. FJ0218

Pingfa Lin [1], Mianmian Shi [2], Feifei Wang [2], Yong Lin [1,*] and Yongbiao Zheng [2,*]

[1] School of Pharmacy, Fujian Health College, Fuzhou 350101, China; linpingfa@163.com
[2] College of Life Sciences, Fujian Normal University, Fuzhou 350117, China; 18135784077@163.com (M.S.); wangfeifei1119@163.com (F.W.)
* Correspondence: lforesty@163.com (Y.L.); yongbiaozheng@fjnu.edu.cn (Y.Z.)

The List of Contents

No.	Content	Page
1	Figure S1 ^{1}H NMR spectra of compound **1**	2
2	Figure S2 ^{13}C NMR spectra of compound **1**	3
3	Figure S3 HR Q-TOF MS data of compound **1**	4
4	Figure S4 ^{1}H NMR spectra of compound **2**	5
5	Figure S5 ^{13}C NMR spectra of compound **2**	6
6	Figure S6 HR Q-TOF MS data of compound **1**	7

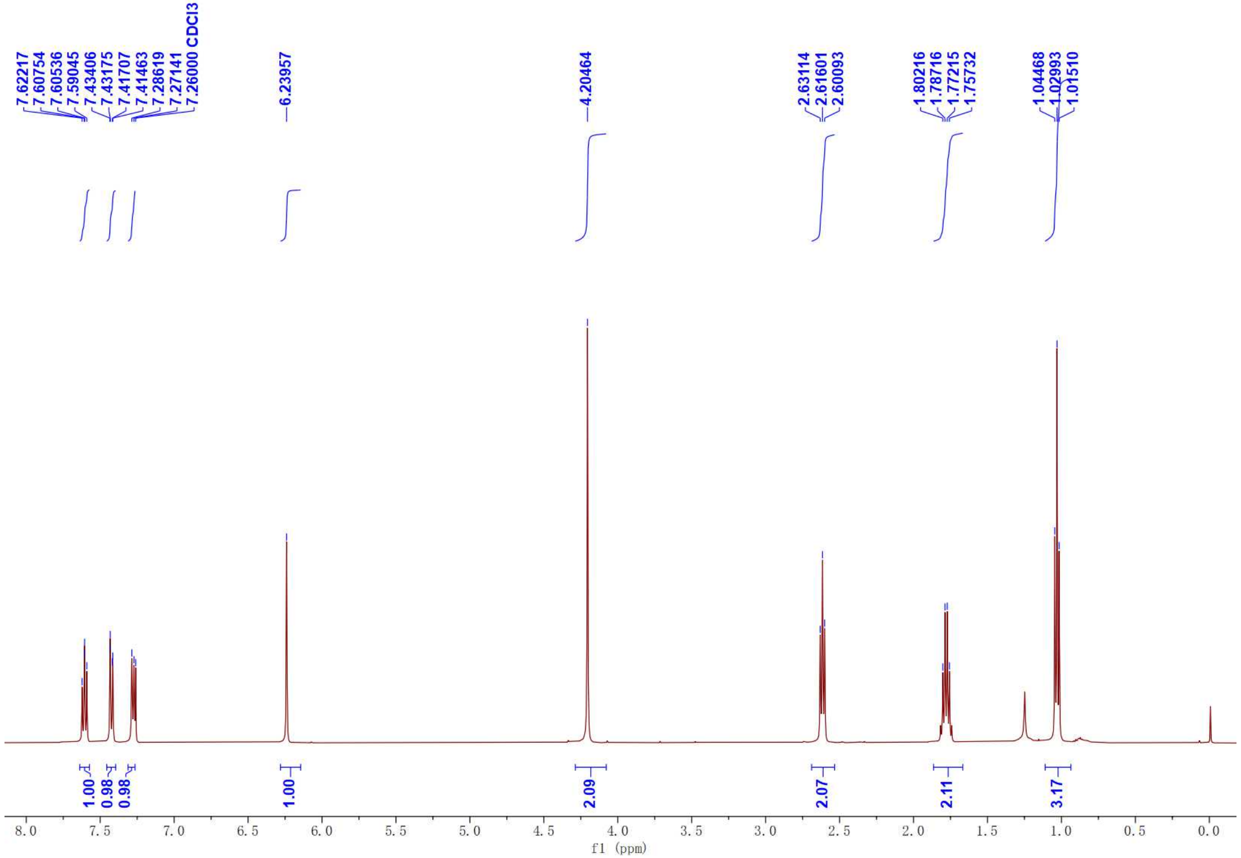

Figure S1 ^{1}H NMR spectra of compound **1** [Recorded at 600 MHz in $CDCL_3$]

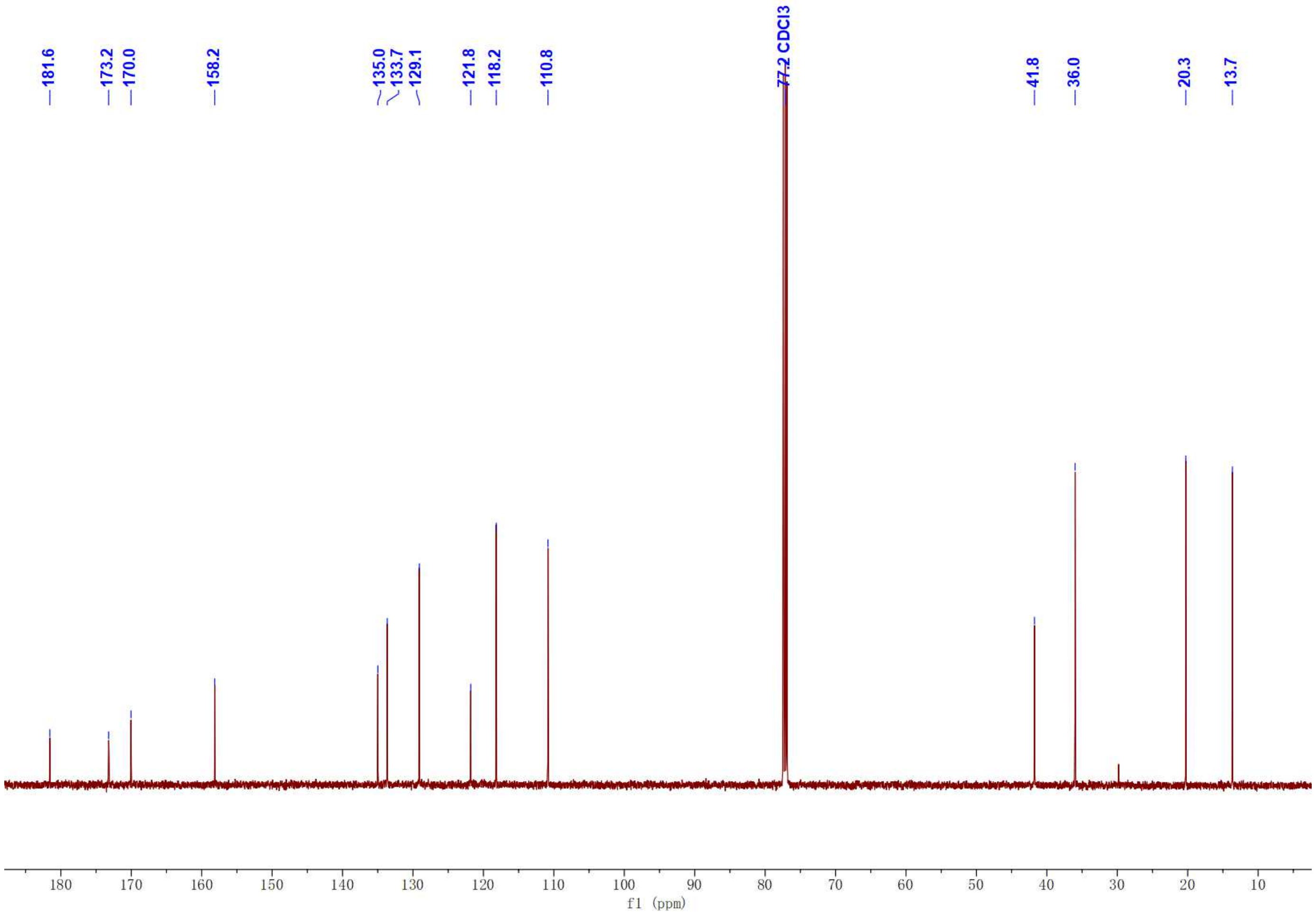

Figure S2 ^{13}C NMR spectra of compound **1** [Recorded at 150 MHz in $CDCL_3$]

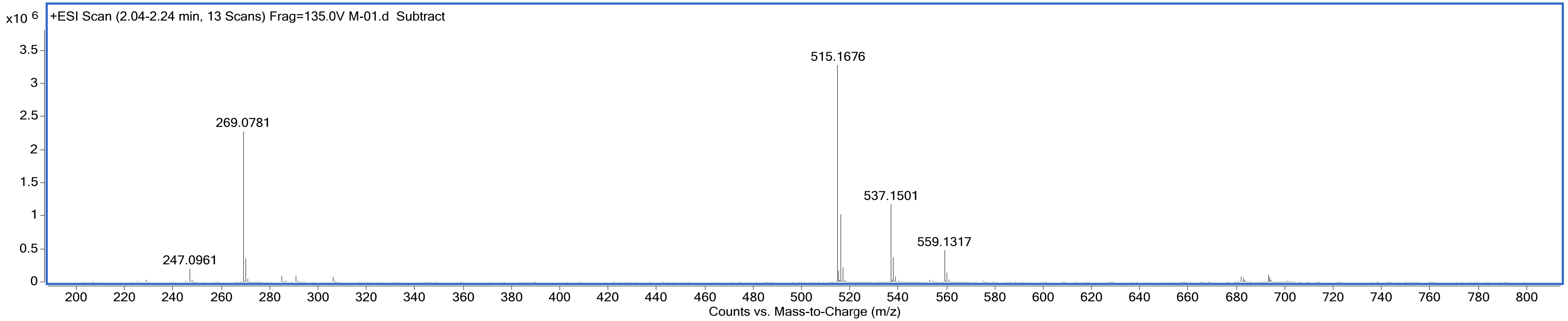

Figure S3 HR Q-TOF MS data of compound **1**.

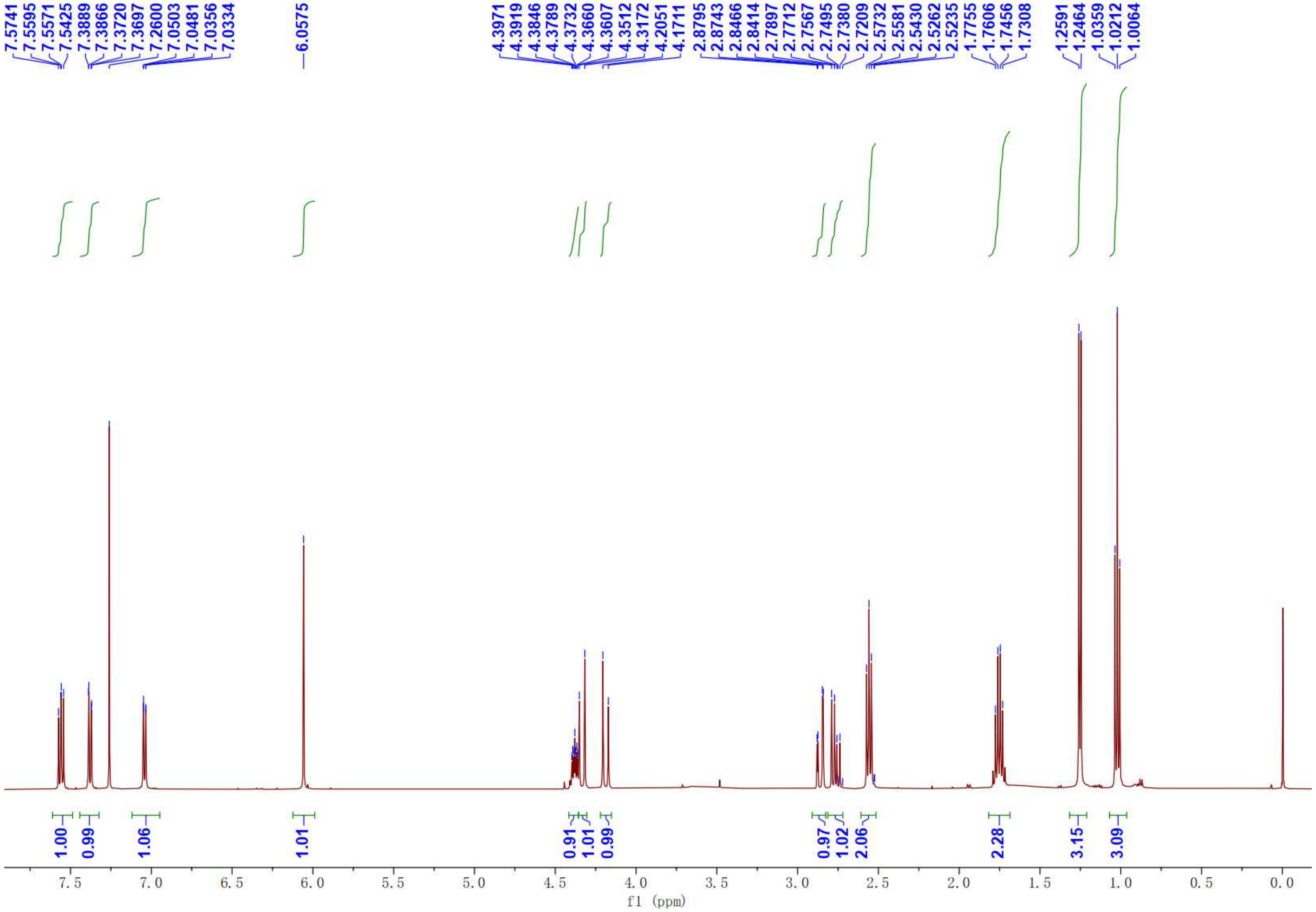

Figure S4 ^{1}H NMR spectra of compound **2** [Recorded at 600 MHz in in $CDCL_3$]

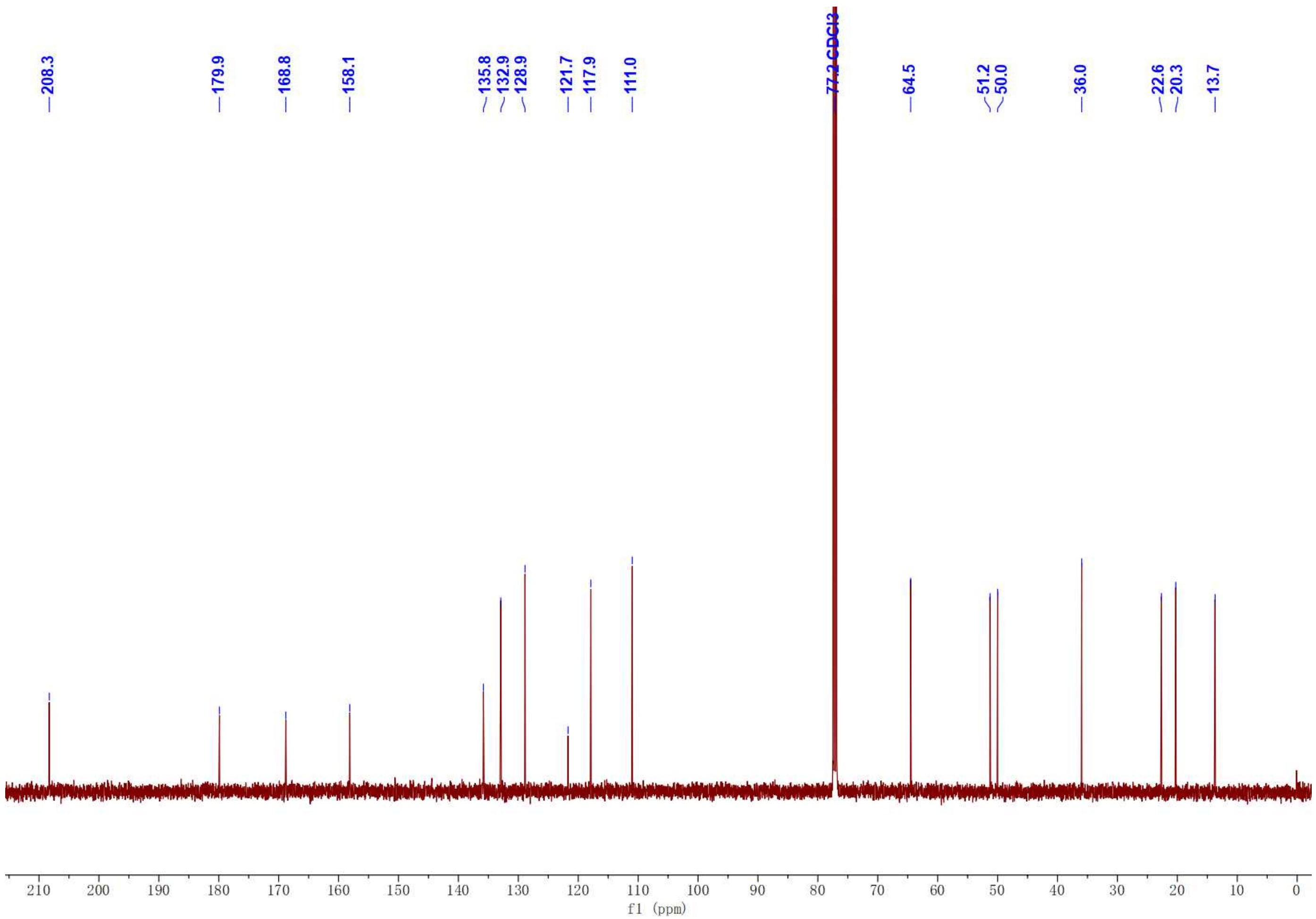

Figure S5 ^{13}C NMR spectra of compound **2** [Recorded at 150 MHz in $CDCL_3$]

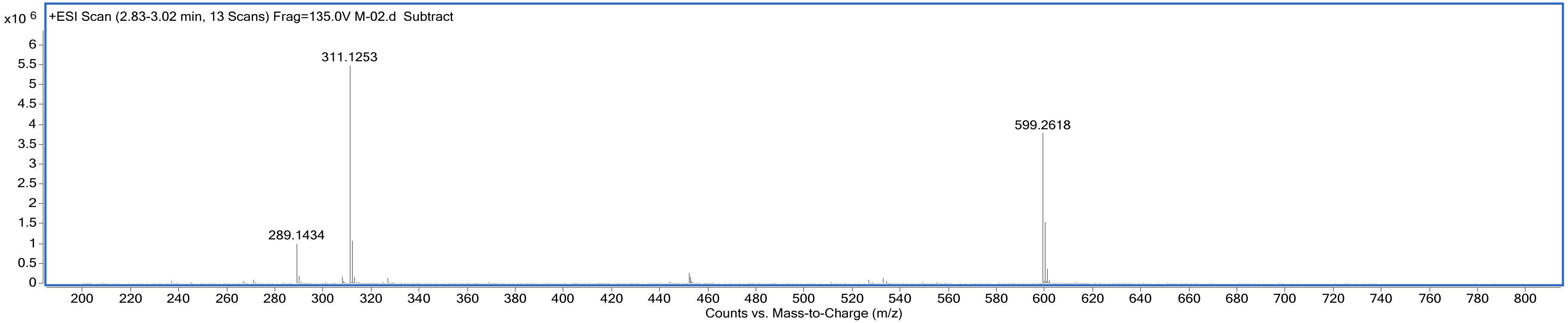

Figure S6 HR Q-TOF MS data of compound 2.
