# Supplementary material for: The α-Glucosidase Inhibition Activities of Phaeochromycins D and E Isolated from Marine Streptomyces sp. FJ0218
Source: Molecules. 2025 Apr 30;30(9):1993. doi: 10.3390/molecules30091993 (PMC12073238; doi:10.3390/molecules30091993)
Supplement: Supplementary file 1 [file molecules-30-01993-s001.zip › File S2.pdf]

# the cif data of phaeochromycin E(CCDC-2383783)

**Table S1 Crystal data and structure refinement for the cif data of phaeochromycin E(CCDC-2383783).**

| | |
|---|---|
| Identification code | the cif data of phaeochromycin E(CCDC-2383783) |
| Empirical formula | $C_{14}H_{14}O_4$ |
| Formula weight | 246.26 |
| Temperature/K | N/A |
| Crystal system | triclinic |
| Space group | P-1 |
| a/Å | 5.1043(2) |
| b/Å | 9.9464(4) |
| c/Å | 12.0965(5) |
| α/° | 95.803(3) |
| β/° | 95.006(3) |
| γ/° | 102.724(3) |
| Volume/Å$^3$ | 592.17(4) |
| Z | 2 |
| $\rho_{calc}$g/cm$^3$ | 1.3810 |
| μ/mm$^{-1}$ | 0.839 |
| F(000) | 260.9 |
| Crystal size/mm$^3$ | N/A × N/A × N/A |
| Radiation | Cu Kα (λ = 1.54184) |
| 2Θ range for data collection/° | 7.4 to 143.52 |
| Index ranges | -6 ≤ h ≤ 6, -12 ≤ k ≤ 12, -14 ≤ l ≤ 14 |
| Reflections collected | 10549 |
| Independent reflections | 2147 [$R_{int}$ = 0.0736, $R_{sigma}$ = 0.0404] |
| Data/restraints/parameters | 2147/0/165 |
| Goodness-of-fit on F$^2$ | 1.065 |
| Final R indexes [I>=2σ (I)] | $R_1$ = 0.0497, w$R_2$ = 0.1365 |
| Final R indexes [all data] | $R_1$ = 0.0525, w$R_2$ = 0.1401 |
| Largest diff. peak/hole / e Å$^{-3}$ | 0.30/-0.34 |

**Table S2 Fractional Atomic Coordinates (×10$^4$) and Equivalent Isotropic Displacement Parameters (Å$^2$×10$^3$) for the cif data of phaeochromycin E(CCDC-2383783). $U_{eq}$ is defined as 1/3 of of the trace of the orthogonalised $U_{IJ}$ tensor.**

| Atom | *x* | *y* | *z* | U(eq) |
|---|---|---|---|---|
| O10 | 7381.2(19) | 5037.6(9) | 8506.2(8) | 23.0(3) |
| O14 | 8684.6(18) | 9400.8(9) | 6076.2(8) | 23.3(3) |
| O15 | 7948.6(19) | 6193.1(10) | 5378.4(8) | 25.0(3) |
| O13 | 6911.9(19) | 8780.5(10) | 4291.9(8) | 25.9(3) |
| C7 | 7673(3) | 5822.4(13) | 6309.3(11) | 20.5(3) |
| C5 | 4566(3) | 7411.3(13) | 6827.9(12) | 20.2(3) |
| C6 | 6076(3) | 6425.1(13) | 7103.2(11) | 19.7(3) |
| C1 | 6054(3) | 6023.2(13) | 8174.0(12) | 20.8(3) |
| C8 | 8964(3) | 4780.8(13) | 6726.8(12) | 22.3(3) |
| C9 | 8803(3) | 4440.8(13) | 7763.8(12) | 22.7(3) |
| C11 | 4324(3) | 7819.7(13) | 5666.3(12) | 21.9(3) |
| C12 | 6880(3) | 8726.3(13) | 5373.3(11) | 20.6(3) |
| C4 | 3215(3) | 7964.4(14) | 7637.3(12) | 22.7(3) |
| C2 | 4704(3) | 6588.3(14) | 8984.8(12) | 23.3(4) |
| C3 | 3301(3) | 7569.5(14) | 8708.1(12) | 24.1(3) |
| C16 | 10086(3) | 3393.8(14) | 8257.4(13) | 25.8(4) |
| C17 | 8162(3) | 1960.9(13) | 8220.1(12) | 25.5(4) |
| C18 | 9651(3) | 933.0(14) | 8681.4(13) | 29.7(4) |

**Table S3 Anisotropic Displacement Parameters ($Å^2×10^3$) for the cif data of phaeochromycin E(CCDC-2383783). The Anisotropic displacement factor exponent takes the form: $-2π^2[h^2a^{*2}U_{11}+2hka^*b^*U_{12}+…]$.**

| Atom | $U_{11}$ | $U_{22}$ | $U_{33}$ | $U_{12}$ | $U_{13}$ | $U_{23}$ |
|---|---|---|---|---|---|---|
| O10 | 21.9(5) | 21.0(5) | 28.3(6) | 8.1(4) | 3.4(4) | 6.4(4) |
| O14 | 20.5(5) | 22.5(5) | 26.0(6) | 3.0(4) | 1.1(4) | 4.3(4) |
| O15 | 24.8(6) | 25.9(5) | 26.7(6) | 8.2(4) | 6.5(4) | 5.4(4) |
| O13 | 22.0(6) | 26.9(6) | 26.9(6) | 0.1(4) | 1.5(4) | 6.9(4) |
| C7 | 16.0(7) | 17.4(6) | 26.2(8) | 1.1(5) | 1.1(5) | 1.6(5) |
| C5 | 15.6(7) | 16.7(6) | 27.1(8) | 1.2(5) | 1.3(5) | 2.8(5) |
| C6 | 15.4(7) | 15.8(6) | 26.6(8) | 1.2(5) | 1.2(5) | 2.4(5) |
| C1 | 16.5(7) | 17.4(6) | 27.9(8) | 3.1(5) | 0.1(5) | 3.8(5) |
| C8 | 18.1(7) | 17.7(6) | 30.7(8) | 4.4(5) | 2.8(6) | 0.8(5) |
| C9 | 17.6(7) | 17.5(6) | 32.0(8) | 3.0(5) | 1.9(5) | 2.1(5) |
| C11 | 17.2(7) | 19.9(7) | 29.1(8) | 5.4(5) | 0.9(6) | 4.5(5) |
| C12 | 19.1(7) | 18.4(6) | 26.5(8) | 8.3(5) | 2.0(5) | 4.1(5) |
| C4 | 17.5(7) | 18.0(6) | 32.8(8) | 4.9(5) | 2.1(6) | 2.8(5) |
| C2 | 21.5(7) | 23.2(7) | 24.3(8) | 3.1(5) | 2.6(6) | 3.3(6) |
| C3 | 20.5(7) | 23.0(7) | 28.7(8) | 5.2(5) | 6.1(6) | -0.2(6) |
| C16 | 21.8(7) | 20.7(7) | 35.5(8) | 6.4(5) | -0.4(6) | 5.4(6) |
| C17 | 24.8(7) | 20.4(7) | 29.9(8) | 4.2(5) | -0.6(6) | 2.9(6) |
| C18 | 33.6(8) | 20.3(7) | 34.9(9) | 7.2(6) | -0.4(7) | 4.0(6) |

**Table S4 Bond Lengths for the cif data of phaeochromycin E(CCDC-2383783).**

| Atom | Atom | Length/Å | Atom | Atom | Length/Å |
|---|---|---|---|---|---|
| O10 | C1 | 1.3807(16) | C6 | C1 | 1.394(2) |
| O10 | C9 | 1.3714(17) | C1 | C2 | 1.389(2) |
| O14 | C12 | 1.2235(17) | C8 | C9 | 1.337(2) |
| O15 | C7 | 1.2298(17) | C9 | C16 | 1.4927(19) |
| O13 | C12 | 1.3159(17) | C11 | C12 | 1.5061(18) |
| C7 | C6 | 1.4770(19) | C4 | C3 | 1.391(2) |
| C7 | C8 | 1.4532(19) | C2 | C3 | 1.382(2) |
| C5 | C6 | 1.4217(19) | C16 | C17 | 1.5365(18) |
| C5 | C11 | 1.5040(19) | C17 | C18 | 1.5227(19) |
| C5 | C4 | 1.383(2) | | | |

**Table S5 Bond Angles for the cif data of phaeochromycin E(CCDC-2383783).**

| Atom | Atom | Atom | Angle/˚ | Atom | Atom | Atom | Angle/˚ |
|---|---|---|---|---|---|---|---|
| C9 | O10 | C1 | 119.10(11) | C9 | C8 | C7 | 122.37(13) |
| C6 | C7 | O15 | 122.92(12) | C8 | C9 | O10 | 122.46(12) |
| C8 | C7 | O15 | 122.39(12) | C16 | C9 | O10 | 111.92(12) |
| C8 | C7 | C6 | 114.67(12) | C16 | C9 | C8 | 125.62(13) |
| C11 | C5 | C6 | 121.33(12) | C12 | C11 | C5 | 113.69(11) |
| C4 | C5 | C6 | 119.05(13) | O13 | C12 | O14 | 123.41(12) |
| C4 | C5 | C11 | 119.57(12) | C11 | C12 | O14 | 123.15(12) |
| C5 | C6 | C7 | 122.89(13) | C11 | C12 | O13 | 113.34(11) |
| C1 | C6 | C7 | 118.95(12) | C3 | C4 | C5 | 121.24(13) |
| C1 | C6 | C5 | 118.16(12) | C3 | C2 | C1 | 118.43(13) |
| C6 | C1 | O10 | 122.37(12) | C2 | C3 | C4 | 120.61(13) |
| C2 | C1 | O10 | 115.18(12) | C17 | C16 | C9 | 113.90(12) |
| C2 | C1 | C6 | 122.46(13) | C18 | C17 | C16 | 110.76(12) |

**Table S6 Hydrogen Atom Coordinates (Å×10$^4$) and Isotropic Displacement Parameters (Å$^2$×10$^3$) for the cif data of phaeochromycin E(CCDC-2383783).**

| Atom | *x* | *y* | *z* | U(eq) |
|---|---|---|---|---|
| H13 | 8325(17) | 9298(16) | 4178.6(14) | 38.9(4) |
| H8 | 9939(3) | 4334.7(13) | 6255.4(12) | 26.7(4) |
| H11a | 3821(3) | 6984.4(13) | 5133.2(12) | 26.3(4) |
| H11b | 2883(3) | 8309.4(13) | 5594.8(12) | 26.3(4) |
| H4 | 2230(3) | 8612.5(14) | 7462.2(12) | 27.2(4) |
| H2 | 4744(3) | 6312.8(14) | 9697.0(12) | 28.0(4) |
| H3 | 2406(3) | 7969.6(14) | 9242.9(12) | 28.9(4) |
| H16a | 11610(3) | 3288.4(14) | 7857.6(13) | 31.0(4) |
| H16b | 10780(3) | 3742.0(14) | 9029.7(13) | 31.0(4) |
| H17a | 6689(3) | 2044.5(13) | 8658.0(12) | 30.5(4) |
| H17b | 7398(3) | 1619.1(13) | 7454.2(12) | 30.5(4) |
| H18a | 11108(14) | 850(9) | 8248(6) | 44.5(6) |
| H18b | 10362(19) | 1258(6) | 9446(3) | 44.5(6) |
| H18c | 8422(6) | 43(3) | 8641(9) | 44.5(6) |

### Experimental

Single crystals of $C_{14}H_{14}O_4$ **[the cif data of phaeochromycin E(CCDC-2383783)]** were **[]**. A suitable crystal was selected and **[]** on a diffractometer. The crystal was kept at N/A K during data collection. Using Olex2 [1], the structure was solved with the Unknown [2] structure solution program using Unknown and refined with the Unknown [3] refinement package using Unknown minimisation.


1.
2.
3.


### Crystal structure determination of [the cif data of phaeochromycin E(CCDC-2383783)]

**Crystal Data** for $C_{14}H_{14}O_4$ ($M$ =246.26 g/mol): triclinic, space group P-1 (no. 2), $a$ = 5.1043(2) Å, $b$ = 9.9464(4) Å, $c$ = 12.0965(5) Å, $\alpha$ = 95.803(3)°, $\beta$ = 95.006(3)°, $\gamma$ = 102.724(3)°, $V$ = 592.17(4) Å$^3$, $Z$ = 2, $T$ = N/A K, μ(Cu Kα) = 0.839 mm$^{-1}$, $Dcalc$ = 1.3810 g/cm$^3$, 10549 reflections measured (7.4° ≤ 2Θ ≤ 143.52°), 2147 unique ($R_{int}$ = 0.0736, $R_{sigma}$ = 0.0404) which were used in all calculations. The final $R_1$ was 0.0497 (I>=2u(I)) and $wR_2$ was 0.1401 (all data).

### Refinement model description

Number of restraints - 0, number of constraints - 23.

Details:

```
1. Fixed Uiso
 At 1.2 times of:
  All C(H) groups, All C(H,H) groups
 At 1.5 times of:
  All C(H,H,H) groups, All O(H) groups
2.a Secondary CH2 refined with riding coordinates:
 C11(H11a,H11b), C16(H16a,H16b), C17(H17a,H17b)
2.b Aromatic/amide H refined with riding coordinates:
 C8(H8), C4(H4), C2(H2), C3(H3)
2.c Idealised Me refined as rotating group:
 C18(H18a,H18b,H18c)
2.d Idealised tetrahedral OH refined as rotating group:
 O13(H13)
```

This report has been created with Olex2, compiled on 2018.05.29 svn.r3508 for OlexSys. Please let us know if there are any errors or if you would like to have additional features.
